# Psychobiographical trends: Untold stories and international voices in the context of social change

**DOI:** 10.1111/jopy.12771

**Published:** 2022-12-05

**Authors:** Claude‐Hélène Mayer

**Affiliations:** ^1^ College of Business and Economics University of Johannesburg Johannesburg South Africa

**Keywords:** extraordinary individuals, minorities, psychobiography, social activism, social agency

## Abstract

**Objective:**

This article describes the increase of publications in the area of psychobiography, thereby analysing the trends in the articles published in the special issue on psychobiography and social agency and activism. It further highlights the specific approaches taken by autobiographers who write psychobiographical accounts of extraordinary individuals and “everyday heroes” in their attempt of creating social impact in specific socio‐cultural groups, societies or even on a global level.

**Method:**

This is a conceptional article which comments on the trends presented in this special issue.

**Results:**

This article presents a discussion of the special issue and its impact.

**Conclusions:**

New trends in psychobiography are discussed and show the way forward for psychobiography.

## 1 UNTOLD STORIES AND INTERNATIONAL VOICES

During recent decades, the art and science of psychobiography have expanded in terms of dynamic change and development in approaches, theories, and methodologies (Kováry, 2011; Mayer et al., 2021). This special issue, edited by Jonathan Adler and Jefferson Singer, presents the work of a diverse group of researchers that span from first‐time practitioners of psychobiography to internationally renowned scholars in the field. These authors have provided a valuable contribution to psychobiography by exploring a range of subjects with shared themes of social agency, social change, and activism.

This special issue offers an opportunity to explore the lives of individuals who may be famous or even relatively unknown in certain scientific, psychobiographical, socio‐cultural, and academic discourses. In the presented articles, these authors recreate the lives of individuals in context (Runyan, 1988, 2013), for example, an Indian reformer and human rights lawyer (Bhatia & Ram), two Black American social activists (Cahill), a Black Lives Matter movement leader (Wilson & Hill), a youth leader and social change agent (Case et al.), and one of the best‐known feminists active in the USA today (Duncan).

The discourses in this special issue foster a vivid counternarrative to the previously dominant discourses and narratives in psychobiography. Previous discourses have often focused on psychoanalytical and WEIRD (Western, educated, industrialized, rich, democratic) individuals, contexts, and perspectives (Mayer et al., 2022), which has contributed to a specific bias in psychobiographical research over the past decades (Mayer, 2022; Mayer & Kelley, 2022).

The authors in this special issue focus on the narratives of outstanding individuals across their lifetimes (Elms, 1994; Fouché & van Niekerk, 2005, 2010), guiding readers into distant and diverse social and cultural metacontexts within India, Australia, China, Russia, South Africa, and the USA, or into extraordinary lives lived in the context of minority groups and discourses. Such meso and micro contexts include LGBTQ+ activists (du Plessis et al.; Weststrate & McLean), Indigenous Native‐Americans (Gone), feminists (Duncan; Josselson), and Black Americans (Cahill; Reynolds et al.) within a dominant, majority‐driven culture. Examining these individuals' lives provides an opportunity to bring new and different discourses into the realm of psychobiography, while also upending traditional hierarchies that specify whose story is “important” and deserves to be told.

Other articles in this special issue give a voice to individuals in extraordinary positions, such as the current American President—whose style of connection with others can be understood through his experiences of loss during his lifetime (McAdams), or a minister's wife who demonstrates an example of a generative identity (Newton & Stewart), or a Chinese business strategist who changed his entire career to focus on social engagement as a teacher (Shu et al.). Other works in the collection of psychobiographical articles include narratives on less well‐known inspiring figures, such as an individual who is driving youth development or medical volunteers during the COVID‐19 pandemic (Case et al.; Nourkova & Gofman).

By mixing the stories of eminent people with narratives of “everyday heroes,” this special issue levels the playing field of psychobiographical subjects and reminds us that all of these profiled individuals possess human frailties, experience intra‐, and interpersonal conflicts, and could not have achieved social change without drawing on networks of relational support. This humanizing of psychobiography is an antidote to the elitism for which it has often been criticized (Elms, 1994).

To accomplish this endeavor, the authors illuminate their subjects through their own personal, social, and cultural lenses and by reflecting on their own biases, which might affect their analyses and interpretations. All of the articles include sections on the researchers' positionality or reflexivity, often describing the challenges experienced in working on and through the material, as well as their choice of discourses regarding the subject and the materials analyzed (Reynolds et al.). These positionality statements and reflections on their methods and conceptual frames add layers of complexity and humility to their psychobiographical studies and belie any claims that their analyses are definitive accounts of the subjects under scrutiny. The authors offer their portrayals as creative invitations rather than ideologically‐driven interpretations.

## 2 PSYCHOBIOGRAPHY DOCUMENTING SOCIAL CHANGE

Psychobiographical studies have previously focused on the lives of individuals in different professions, such as politicians, actors, singers, and psychologists (e.g., Kováry, 2011). But the area of psychobiographies focusing on social change agents is still limited (de la Sablonnière, 2017).

This special issue enriches previous psychobiographical research by contributing to the exploration of the lives of social activists and agents from different perspectives. An example is the life of Babasaheb Ambedkar, who was a social reformer and human rights lawyer in India. Ambedkar also made a significant contribution to constructing the Indian constitution (Bhatia & Ram). Psychobiography in the context of exploring the lives of social agents can offer insights into social change as a socio‐historical phenomenon, but also achieve a deeper understanding of specific socio‐cultural and contextual discourses, which are constructed during significant intervals in the individuals' lives. In some of these social agency‐ and activism‐related psychobiographies, the authors dismantle the overemphasis on the individual agent and the notion that one individual is the major fulcrum of social change. In so doing, these authors highlight how socio‐cultural majority perspectives and systemic influences can assert tremendous power and easily become oppressive. The subjects of these psychobiographies often resist this pressure, but they seldom do so or succeed in isolation. Across the range of biographies, we see the crucial role of family, mentors, colleagues, and larger social movements in launching the subjects into life‐changing and society‐changing action.

Accordingly, the editors of this special issue give psychobiography a new slant, not only with new theoretical and conceptual implications but also with practical relevance for social movements and leaders now and in the future. Several of the articles tap into new understandings of how social agents and activists become motivated during their lives, the key situations they experience, the resilience and personality characteristics needed, the integration of personal motivations and attitudes, the kinds of narrative arcs, and the impact of socio‐cultural and historical contexts on these motivations. Furthermore, it is worthy to mention that one of the trends in these psychobiographies is to refrain from exploring the entire life history of psychobiographies, instead selecting specific key life situations and turning points for in‐depth analysis from different theoretical perspectives.

The narratives presented in this special issue respond to Carlson's (1971) still‐valid provocation from a half‐century ago: “Where is the person in personality research?” The authors and editors of the special issue place the person and their motivations, intentions, and conscious longing for and dreaming of social change right at the nexus of personality research and social movement psychology. However, in doing so, they also embrace a focus on the different social and cultural contexts that shape these individuals' identities and that reflect socio‐political systems and their dynamics. In addition, the implications of various socio‐historical movements on the lives of individuals are explored—movements such as decolonization, the politics of resistance, civil rights movements in various socio‐cultural and political contexts, responses to state terrorism, LGTBQ+ movements, Black Lives Matter, and Black radical freedom movements, and the overall efforts toward a critical consciousness in academia. Given the range of human experience covered, psychological professional contexts and therapeutic approaches can make use of these psychobiographical analyses in therapeutic practices, such as existential meaning therapy, systemic, family, or arts therapy, humanistic approaches to treatment, and general consciousness‐raising.

## 3 RECENT PSYCHOBIOGRAPHICAL DEVELOPMENTS

This special issue represents the trend of expanding the field of psychobiography within psychological contexts (Wegner, 2020) and spreading the field to include diverse and hybrid narratives from all over the world. These developments are reflected, for example, in increasing numbers of articles, book chapters, and books published in the past two decades, dealing with psychobiography, since the 2005 publication of Schultz's *Handbook of Psychobiography*. Several publications (Adler, 2018; Mayer & Kovery, 2019; Mayer et al., 2021, 2022) have built on the pioneering work of Runyan (1988), Schultz (2005), and McAdams (1988, 1999), expanding theories and methodologies, as well as specific topics within psychobiography such as ethics (Ponterotto, 2014; Ponterotto et al., 2015; Ponterotto & Reynolds, 2017). They have thereby reinforced a culturally and socio‐politically sensitive way of seeing and interpreting the world and have created a new understanding of the larger historical and structural influences of societies in the context of power relations and power struggles (e.g., Weststrate & McLean, 2022, this issue). These authors' approaches to psychobiography highlight the following fact: in order to understand the identity development of an individual, culture, and history need to be comprehended with a transactional approach to seeing the world and integrating different theoretical lenses to capture the complexity of identity development. Weststrate and McLean's article in this special issue is a particularly eloquent plea to recognize intersectionality in identities and histories, to see and acknowledge their complexities, and to counteract the reductionism that has often been criticized in psychobiographies (e.g. Elms, 1994).

Besides a growing number of publications published in the USA, the number of psychobiographies from the South African context (Fouché, 2015; Fouché & van Niekerk, 2010) and Eastern Europe (Citlak, 2021; Kováry, 2011) has grown. The formation of a psychobiography forum of international standing has also taken place over the past 2 years, initiated by:

- Paul H. Elovitz, Ph.D., Historian, Research Psychoanalyst, Associate Professor at Ramapo College of New Jersey, Director of the Psychohistory Forum, and Editor of the journal *Clio's Psyche*.
- Inna Rozentsvit, M.D., Ph.D., Founder & Neuropsychoeducator, NeuroRecovery Solutions and Programs Director, Scientific Faculty Member, Co‐Founder of the Parent–Child Development and The Neuropsychoanalysis Programs, Object Relations Institute for Psychoanalysis and Psychotherapy, NYC, USA.
- Claude‐Hélène Mayer, Ph.D. Professor of Industrial and Organizational Psychology, University of Johannesburg, South Africa, Systemic Family Therapist and Intercultural Mediator.

This psychobiographical group, as part of the psychohistorical forum, aims to foster research in psychobiography and establish international cooperation in the field of psychobiography. The intention is to build a worldwide cooperative community of psychobiographers and psychohistorians that will contribute to the development of the psychobiographical sub‐discipline and its international and intercultural standing.

This special issue represents a new trend in psychobiography that explores specific themes in the lives of individuals, their deeper motivations, and their interconnectedness on micro, meso, and macro levels, thereby emphasizing the dynamic interchange of socio‐political contexts and individual agency. Out of this nexus, one finds rich implications for social agency and activism developed in real‐world scenarios on a global level.

## AUTHOR CONTRIBUTIONS

The article has been fully written by the author.

## CONFLICT OF INTEREST

The authors report no conflicts of interest.

## ETHICAL APPROVAL

Ethical considerations have been applied in this research.
